# 41538 Characterizing Opioid Overdose Hotspots for Targeted Overdose Prevention and Treatment

**DOI:** 10.1017/cts.2021.619

**Published:** 2021-03-31

**Authors:** Elizabeth A. Samuels, William Goedel, Lauren Conkey, Jennifer Koziol, Sarah Karim, Rachel P. Scagos, Lee Ann Jordison Keeler, Rachel Yorlets, Neha Reddy, Sara Becker, Roland Merchant, Brandon D. L. Marshall

**Affiliations:** 1 Brown Emergency Medicine; 2 Brown University School of Public Health; 3 Rhode Island Department of Health; 4 Warren Alpert Medical School of Brown University; 5 Brigham and Women’s Hospital

## Abstract

ABSTRACT IMPACT: Identifying factors associated with opioid overdoses will enable better resource allocation in communities most impacted by the overdose epidemic. OBJECTIVES/GOALS: Opioid overdoses often occur in hotspots identified by geographic and temporal trends. This study uses principles of community engaged research to identify neighborhood and community-level factors associated with opioid overdose within overdose hotspots which can be targets for novel intervention design. METHODS/STUDY POPULATION: We conducted an environmental scan in three overdose hotspots’‘ two in an urban center and one in a small city’‘ identified by the Rhode Island Department of Health as having the highest opioid overdose burden in Rhode Island. We engaged hotspot community stakeholders to identify neighborhood factors to map within each hotspot. Locations of addiction treatment, public transportation, harm reduction programs, public facilities (i.e., libraries, parks), first responders, and social services agencies were converted to latitude and longitude and mapped in ArcGIS. Using Esri Service Areas, we will evaluate the service areas of stationary services. We will overlay overdose events and use logistic regression identify neighborhood factors associated with overdose by comparing hotspot and non-hotspot neighborhoods. RESULTS/ANTICIPATED RESULTS: We anticipate that there will be differing neighborhood characteristics associated with overdose events in the densely populated urban area and those in the smaller city. The urban area hotspots will have overlapping social services, addiction treatment, and transportation service areas, while the small city will have fewer community resources without overlapping service areas and reduced public transportation access. We anticipate that overdoses will occur during times of the day when services are not available. Overall, overdose hotspots will be associated with increased census block level unemployment, homelessness, vacant housing, and low food security. DISCUSSION/SIGNIFICANCE OF FINDINGS: Identifying factors associated with opioid overdoses will enable better resource allocation in communities most impacted by the overdose epidemic. Study results will be used for novel intervention design to prevent opioid overdose deaths in communities with high burden of opioid overdose.

